# Supplementary material for: Population dynamics and entrainment of basal ganglia pacemakers are shaped by their dendritic arbors
Source: PLoS Comput Biol. 2019 Feb 7;15(2):e1006782. doi: 10.1371/journal.pcbi.1006782 (PMC6382172; doi:10.1371/journal.pcbi.1006782)
Supplement: S1 Appendix — (DOCX) [file pcbi.1006782.s004.docx]

**S1 Appendix**

#adjust defaults for xpp internal variables

@ MAXSTOR=40000

@ YP=delta

@ XP=psi

@ TOTAL=.25

@ DT=0.001

@ BOUND=100000

@ XHI=1

@ YLO=-.2

@ YHI=.2

@ METH=qual

@ BACKGROUND=white

@ BUT=Quit:fq

# phase response function

p theta=.75

prc(x)=x*heav(theta-x)*heav(x)/theta+(1-x)*heav(x-theta)*heav(1-x)/(1-theta)+(x-1)*heav(1+theta-x)*

heav(x-1)/theta+(2-x)*heav(x-theta-1)*heav(2-x)/(1-theta)+(x+1)*heav(theta-x-1)*heav(x+1)/theta+(-x)*heav(1+x-theta)*heav(-x)/(1-theta)

# diff equ

dpsi/dt=0

dphi/dt=g+eps*prc(phi)*cos(2*pi*(f*t+psi))

aux delta=g*t-1

# ic

i phi=0

i psi=0

# param

p g=7,f=7,eps=-5

# eps is negative so that phase 0 is at the trough of the cosine

# To Run do n(U)merics (P)oincare map (S)ection: variable: PHI Section: 1 Direction: 1 Stop on sect: Y

# then do esc (I)ntegrate (R)ange Range over: PSI Steps:100 Start: 0 End: 1 Reset Storage: N

d
